# Burden of illness among patients with asthma prescribed inhaled corticosteroids/long-acting β_2_-agonists

**DOI:** 10.1038/s41533-024-00402-w

**Published:** 2025-02-26

**Authors:** Shiyuan Zhang, Alexandrosz Czira, Julia Harley, Kieran J. Rothnie, Lauren Lee, Mark Small

**Affiliations:** 1 GSK, R&D Global Medical, Collegeville, PA USA; 2 GSK, R&D Global Medical, Brentford, Middlesex UK; 3 Adelphi Real World, Bollington, Cheshire, UK

**Keywords:** Asthma, Epidemiology, Outcomes research

## Abstract

Inhaled corticosteroids (ICS) plus long-acting β_2_-agonists (LABA) are recommended for maintenance-only or maintenance and reliever therapy (MART) in patients with asthma. However, real-world data on ICS/LABA as maintenance-only or MART are limited. This study characterized clinical, economic, and humanistic burdens of asthma in Canada, China, Europe, Japan, and the US, using data collected from patients and physicians via a cross-sectional survey (Asthma Disease Specific Programme). Patients were ≥18 years of age with physician-confirmed asthma and receiving fixed-dose ICS/LABA for ≥3 months. Mean physician-reported symptom-free days over the past 30 days ranged from 10.1–20.6 days, and 31.5–34.6% of ICS/LABA users self-reported not well-controlled asthma. SABA co-prescription was reported in 8.8–67.8% of patients. These findings highlight the continued disease burden among ICS/LABA users, with the high level of SABA co-prescription indicating potentially inappropriate prescribing of ICS/LABA as MART or detrimental reliance on SABA medication in addition to MART.

## Introduction

Asthma is a chronic, heterogeneous respiratory disease, characterized by symptoms of airflow limitation, wheezing, shortness of breath and cough, with an incidence ranging from 1 to 29% across various countries^1^. Uncontrolled asthma is associated with considerable burden of disease, including impaired lung function and exacerbations, high healthcare resource utilization (HCRU) and costs, and impaired health-related quality of life (HRQoL)^2^. The goal of asthma management is, therefore, to achieve and maintain control of symptoms and activity levels, reduce airflow limitation, and limit the risk of future exacerbations^1^.

The Global Initiative for Asthma (GINA) 2024 report recommends a low-dose inhaled corticosteroid/long-acting β_2_-agonist (ICS/LABA) combination (budesonide/formoterol or beclomethasone/formoterol) as needed at GINA treatment Steps 1–2, and low–medium-dose ICS/LABA as maintenance and reliever therapy (MART) at GINA treatment Step 3 and above^1^. This approach is recommended as a preferred option over maintenance treatment with ICS/LABA plus as-needed short-acting β_2_-agonist (SABA)^1^. This is based on evidence from clinical trials that ICS/LABA prescribed as MART can achieve the same or improved asthma control compared with ICS/LABA as maintenance therapy plus as-needed SABA^3,4^, as well as recent reports from observational studies indicating that a large proportion of patients with asthma are highly reliant on or overuse SABA^5,6^. Furthermore, despite limited real-world data for ICS/LABA prescribed as MART, a 2022 study analyzing the electronic medical records of Korean patients with asthma found that the risk of exacerbations and overall corticosteroid requirements were lower in patients with ICS/LABA MART versus those receiving ICS/LABA plus SABA^7^.

Nonetheless, asthma still presents a considerable disease burden, even with treatment availability and the advent of global asthma management guidelines. Notably, 28–80% of patients are estimated to have asthma that is poorly controlled despite treatment with ICS/LABA, prescribed as either fixed daily maintenance therapy or MART^8,9^. In addition, a recent survey-based study found that, among patients with asthma in the United States (US) who reported moderate/high adherence to ICS/LABA, 81% continued to experience symptoms, 50% had inadequately controlled asthma, and work impairment and HCRU were considerable^10^.

Given the scarcity of real-world data on the use of ICS/LABA as MART relative to maintenance-only therapy despite GINA guideline recommendation of MART at Step 3 and above^1^, the primary objective of this study was to describe the clinical, economic and humanistic burden of patients with asthma prescribed ICS/LABA overall (as a reliever, maintenance-only therapy, or MART), ICS/LABA as MART or ICS/LABA as maintenance-only therapy, across Canada, China, Europe, Japan, and the US. The secondary objective was to describe the characteristics (demographic, concomitant medications and asthma-related) of these patients, including the types of physicians prescribing these regimens and physician-reported reasons for prescribing.

## Methods

### Study design

**Fig. 1:**
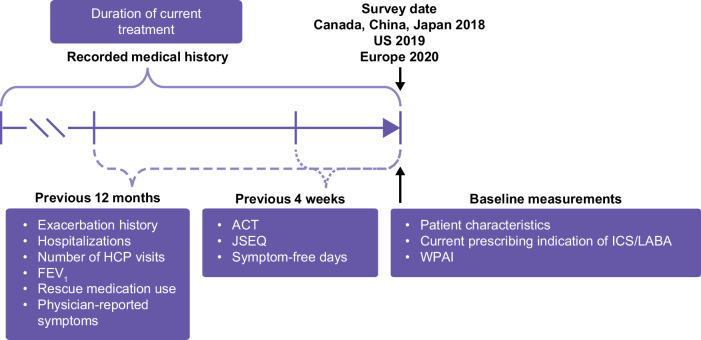
Study design. ACT Asthma Control Test, FEV_1_ forced expiratory volume in 1 s, HCP healthcare professional, ICS inhaled corticosteroids, JSEQ Jenkins Sleep Evaluation Questionnaire, LABA long-acting β_2_-agonist, US United States, WPAI Work Productivity and Activity Impairment.

This retrospective cohort study was conducted among patients with asthma who consulted with primary care physicians (PCPs), pulmonologists or allergists in Canada, China, Europe, Japan, and the US (2018–2020), using data previously captured as part of the cross-sectional Adelphi Real World Asthma Disease Specific Programme (DSP) survey, a point-in-time physician and patient survey conducted to provide impartial observations of real-world clinical practice^11,12^. Data for Europe were available from a representative sample of 5 countries (France, Germany, Italy, Spain, and the United Kingdom [UK]). The DSP survey date was defined as the baseline period (Fig. 1).

### Study population

Adult patients (≥18 years of age) with a physician-confirmed diagnosis of asthma who visited their physician in a routine care setting were eligible for inclusion in the DSP survey and a consecutive patient sample (the next 4–5 patients seen by the treating physician) was used. For this study, eligible patients were receiving any fixed ICS/LABA therapy (any dosage), with or without maintenance or reliever inhaler medication for ≥3 months at the survey date. ICS/LABA MART prescribing was identified via a physician-completed tick box. Patients were excluded if they had a physician-confirmed co-diagnosis of chronic obstructive pulmonary disease or were already participating in a clinical trial at the survey date.

Eligible physicians included in the DSP survey were hospital-based specialists, PCPs, pulmonologists or allergists who were personally responsible for treatment decisions and saw ≥3 patients with asthma each month. Physicians were screened by speciality and geography to provide a geographically diverse sample distribution across each country.

### Data collection and assessments

Data collection was completed from July 2018 to December 2018 in Canada, China, and Japan; from July 2019 to December 2019 in the US; and from January 2020 to June 2020 in Europe. All information collected during the baseline period was from available medical histories or a patient recall period in the preceding 12 months; no follow-up data were collected (Fig. 1). Medical assessments conducted during routine medical practice were recorded by the physician as part of the survey. Patient-reported outcome (PRO) instruments, including the Work Productivity and Activity Impairment (WPAI) questionnaire, Asthma Control Test (ACT) questionnaire, Jenkins Sleep Evaluation Questionnaire (JSEQ), and EuroQol 5 dimensions (EQ-5D) questionnaire were voluntarily self-completed by patients as part of a large patient questionnaire. The use of these instruments differed between countries due to availability and licensing agreements at the survey date (WPAI available in the US, Europe, and China; ACT and JSEQ available in the US and Europe).

The characteristics of ICS/LABA users (demographic, concomitant medications, treatment adherence, and asthma-related), including the types of physicians prescribing these regimens (primary care vs. specialty) and physician-reported reasons for prescribing, were assessed using current data and available medical histories at baseline. Physician-reported patient adherence was measured using a single choice question in the patient record form: “How adherent is this patient with their treatment regimen in terms of the number of times they take their asthma treatment as prescribed in the last 12 months?” The question was scored using a 5-point scale from ‘not at all adherent’ to ‘completely adherent’. The clinical burden was assessed using lung function data (pre-bronchodilator forced expiratory volume in 1 second [FEV_1_] % predicted), clinical characteristics, exacerbation frequency, and reliever medication use from the preceding 12 months. Economic burden was assessed based on HCRU in the past 12 months (number of Health Care Professional [HCP] visits and hospitalizations due to asthma) and WPAI at baseline. The WPAI questionnaire is a 6-item quantitative measure of health-related work productivity and activity impairment over the past 7 days; overall, higher outcome scores reflect greater impairment due to health problems^13^. The humanistic burden was assessed based on the number of symptom-free days in the previous 30 days, frequency of physician-reported most troublesome symptoms in the last 12 months, and HRQoL from the previous 4 weeks; HRQoL assessments included the ACT and JSEQ. The ACT questionnaire is a 5-item quantitative measure of asthma control that assesses symptoms, reliever medication use, and the impact of asthma on day-to-day functioning using a 5-point Likert scale^14^. Scores are summed ranging from 5–25, with higher scores indicating better asthma control. The JSEQ is a 4-item quantitative measure of sleep disturbance over the previous 4 weeks, scored using a 6-point alternative response scale based on the number of days sleep is disturbed (0: not at all; 1: 1–3 days; 2: 4–7 days; 3: 8–14 days; 4: 15–21 days; and 5: 22–28 days)^15^. Scores are summed from 0–20, with higher scores indicating greater sleep disturbance. The EQ-5D is a two-part patient-completed health questionnaire that measures HRQoL via a 5-dimensional utility score (mobility, self-care, usual activities, pain/discomfort, and anxiety/depression; total score summed from 0 to 1) and a visual analog scale (VAS; score summed from 0 to 100); higher values indicate better HRQoL^16^.

### Data analyses

All analyses were performed by Adelphi Real World. Descriptive statistics for each population were generated using IBM SPSS Data Collection Survey Reporter, no comparative statistical analyses were carried out. Continuous variables were presented as the number of responses, mean, and standard deviation (SD). Categorical variables were presented as the frequency and percentage of patients within each response. Missing data were not included when calculating percentages and missing values were not imputed. Data for each outcome were collected for all ICS/LABA users and further stratified by MART and maintenance subpopulations.

### Ethical approval

Western International Review Board methodological exemption was obtained for Canada (1-10998882-1), Europe and the US (1-1236810-1), and Japan (1-1099576-1). The Asthma DSP in China was conducted according to codes of conduct laid out by the European Pharmaceutical Marketing Research Association (EPHMRA) guidelines and the Chinese Marketing Research Association. This study complied with all applicable laws regarding subject privacy. No direct subject contact or primary collection of individual human subject data occurred, and no patient identifiers are reported.

## Results

### Study population

**Fig. 2:**
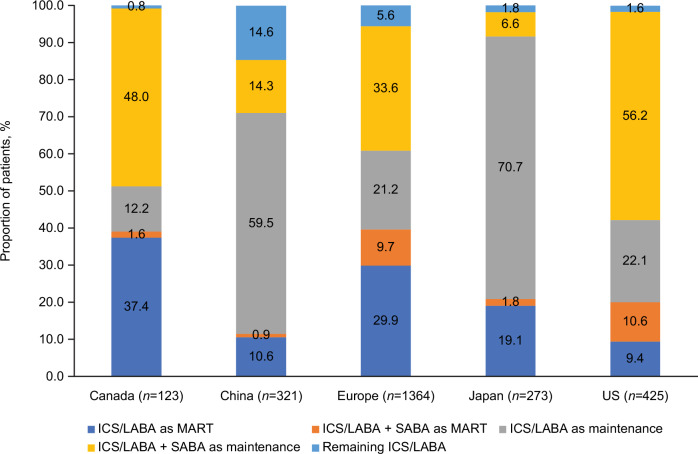
ICS/LABA prescription by region. ICS inhaled corticosteroid, LABA long-acting β_2_-agonist, MART maintenance and reliever therapy, SABA short-acting β_2_-agonist, US United States.

A total of 2506 patients met the inclusion criteria and were prescribed ICS/LABA medication for ≥3 months in Canada (123/2,506 [4.9%]), China (321/2,506 [12.8%]), Europe (1,364/2,506 [54.4%]), Japan (273/2,506 [10.9%]), and the US (425/2,506 [17.0%]) (Supplementary Fig. 1). When stratified by subpopulations, most ICS/LABA users were prescribed ICS/LABA as maintenance-only therapy in all 5 regions (Canada, 74/123 [60.2%]; China, 237/321 [73.8%]; Europe, 747/1,364 [54.8%]; Japan, 211/273 [77.3%]; and the US, 333/425 [78.4%]) (Fig. 2). Among ICS/LABA MART users, patients in Canada, China, Europe, and Japan were predominantly prescribed ICS/formoterol as MART as recommended by GINA; in contrast, most patients in the US were prescribed an ICS/LABA therapy not recommended for MART by GINA (i.e., non-ICS/formoterol combinations) (Supplementary Fig. 1).

#### Patient demographics and clinical characteristics

For ICS/LABA users across all regions, mean (SD) age ranged from 43.4 (16.4) to 53.0 (17.5) years, most patients were female (51.7–55.4%), and most had never smoked (58.2–76.6%) (Table 1). In all five regions, the mean Charlson Comorbidity Index (CCI) score was 0.1 (SD range: 0.4–0.5). Allergic rhinitis was the most common comorbidity in China (50.8%), Europe (39.9%), and the US (52.2%), while hypertension was the most common comorbidity in Canada (23.6%) and Japan (23.1%) (Table 1). Most patients were in GINA treatment Step 4 in each region, with China having the lowest proportion of patients in GINA treatment Step 5 (0.3%) (Table 2).

**Table 1:** Patient demographics at baseline for all ICS/LABA users.

	All ICS/LABA users				
	Canada (*N* = 123)	China (*N* = 321)	Europe (*N* = 1364)	Japan (*N* = 273)	US (*N* = 425)
**Age**	*n* = 122	*n* = 265	*n* = 1364	*n* = 272	*n* = 425
Mean (SD)	44.7 (16.5)	44.1 (13.6)	43.4 (16.4)	53.0 (17.5)	46.2 (16.8)
**Sex,** **%**					
Female	52.9	51.7	55.4	55.3	54.8
**BMI**	*n* = 123	*n* = 317	*n* = 1364	*n* = 273	*n* = 425
Mean (SD)	27.2 (7.4)	22.5 (2.2)	25.70 (4.7)	24.30 (24.8)	27.90 (6.3)
**Employment status, %**					
Working full time	56.9	67.0	56.7	42.9	59.3
Working part time	14.6	2.2	8.3	11.4	7.8
Long term sick leave	1.6	0.3	1.2	0.4	0.2
Homemaker	4.9	4.1	7.3	22.0	7.8
Student	7.3	0.9	11.6	3.3	6.8
Retired	11.4	17.1	11.4	8.4	14.4
Unemployed	3.3	7.8	2.6	5.9	3.3
Don’t know	0.0	0.6	1.0	5.9	0.5
**Smoking status, %**	*n* = 123	*n* = 320	*n* = 1364	*n* = 273	*n* = 425
Current smoker	11.4	1.6	13.3	6.6	5.7
Ex-smoker	21.1	20.6	21.1	24.2	17.2
Never smoked	66.7	76.6	63.4	58.2	75.8
Unknown	0.8	1.3	2.2	11.0	1.4
**Physician specialty, %**^a^					
PCP	79.7	N/A	52.9	66.3	51.3
Pulmonoogist/respiratory specialist	20.3	N/A	37.5	33.7	32.9
Allergist	0.0	N/A	9.6	0.0	15.8
Hospital-based specialist	N/A	100.0	N/A	N/A	N/A
**Charlson comorbidity index**					
Mean (SD)	0.1 (0.5)	0.1 (0.4)	0.1 (0.5)	0.1 (0.5)	0.1 (0.5)
**Comorbidities, %**^b^					
Allergic rhinitis	N/A	50.8	39.9	N/A	52.2
Anxiety	18.7	2.8	11.0	1.8	17.7
Arthritis	8.1	6.9	2.4	1.5	9.9
Atopic dermatitis	N/A	2.5	11.6	N/A	8.0
Chronic rhinosinusitis	N/A	11.8	9.9	N/A	16.2
Depression	12.2	2.8	5.6	1.5	10.8
Diabetes	7.3	11.5	4.3	4.4	10.6
Elevated cholesterol	9.8	6.2	7.9	13.6	21.2
GERD	12.2	0.6	6.8	5.5	13.4
Hypertension	23.6	19.3	18.1	23.1	32.7
Nasal polyps	N/A	4.7	7.5	N/A	4.5
Obesity	8.1	1.6	6.4	2.6	11.8
None of the above	N/A	9.4	30.5	N/A	20.9
None	43.1	N/A	N/A	55.0	N/A

Data for variables indicated as N/A were not collected.

*BMI* body mass index, *GERD* gastroesophageal reflux disease, *ICS* inhaled corticosteroid, *LABA* long-acting β_2_-agonist, *N/A* not available, *PCP* primary care physician, *SD* standard deviation, US United States.

^a^Optional responses for physician speciality in Canada, Europe, Japan, and the US included PCPs, pulmonologists/respiratory specialists, and allergists; in China, physician speciality response options were limited to hospital-based specialists.

^b^Only comorbidities reported for ≥5% of patients are presented.

**Table 2:** Clinical characteristics and exacerbations at baseline for all ICS/LABA users.

	All ICS/LABA users				
	Canada (*N* = 123)	China (*N* = 321)	Europe (*N* = 1364)	Japan (*N* = 273)	US (*N* = 425)
**Most recent pre-bronchodilator FEV**_**1**_ **predicted score** **(%)**	*n* = 42	*n* = 79	*n* = 811	*n* = 55	*n* = 215
Mean (SD)	91.2 (109.0)	55.9 (9.9)	74.2 (18.9)	78.8 (11.9)	72.4 (19.5)
**Most recent blood eosinophil count (cells/µl)**	*n* = 51	*n* = 41	*n* = 577	*n* = 184	*n* = 139
Mean (SD)	337.1 (240.5)	235.1 (349.3)	355.3 (233.3)	434.9 (280.8)	364.9 (294.0)
**FeNO test result, %**	*n* = 2	*n* = 210	*n* = 259	*n* = 56	*n* = 27
Low	0.0	38.6	18.5	10.7	25.9
Intermediate	100.0	57.1	49.4	30.4	25.9
High	0.0	4.3	32.1	58.9	48.2
**GINA**^a,b^ **treatment** **Step***,* *%*	*n* = 122	*n* = 321	*n* = 1294	*n* = 268	*n* = 416
Step 1	27.9^c^	0.0	0.2	11.2^c^	0.0
Step 2	27.9^c^	0.0	0.0	11.2^c^	0.0
Step 3	12.3	29.9	33.1	13.8	17.6
Step 4	35.3	69.8	43.7	44.0	60.3
Step 5	24.6	0.3	22.8	31.0	22.1
**Exacerbations in the last 12 months (any severity), %**					
0	56.9	89.4	57.4	79.5	52.7
1	21.1	6.9	22.1	10.3	27.5
≥2	22.0	3.7	20.5	10.3	19.8
Mean (SD)	0.9 (1.5)	0.2 (0.6)	0.8 (1.2)	0.5 (1.3)	0.9 (1.4)
**Exacerbations in the last 12 months resulting in OCS course and/or ER visit and/or hospitalization, %**	*n* = 123	*n* = 321	*n* = 1364	*n* = 273	*n* = 425
0	62.6	91.9	66.1	82.1	63.1
1	17.9	5.9	16.3	9.5	21.9
≥2	19.5	2.2	17.7	8.4	15.1
Mean (SD)	0.7 (1.3)	0.1 (0.4)	0.6 (1.1)	0.4 (1.2)	0.6 (1.1)

Data for variables indicated as N/A were not collected.

*ER* emergency room, *FeNO* fractional exhaled nitric oxide, *FEV*_1_ forced expiratory volume in 1 s, *GINA* Global Initiative for Asthma, *ICS* inhaled corticosteroid, *LABA* long-acting β_2_-agonist, *N/A* not available, *OCS* oral corticosteroid, *SD* standard deviation, *US* United States.

^a^GINA 2019 treatment Step reported in Canada and Japan^28^; GINA 2020 treatment Step reported in China, Europe, and the US^29^; ^b^Treatment prescribed not currently recommended by GINA was undefined for Canada and China; the proportion of all ICS/LABA users in the remaining regions was 0.3 (Europe), 0.0 (Japan), and 0.0 (the US).

^c^Data for GINA Steps 1 and 2 were combined; this population includes those who were prescribed ICS with formoterol.

#### Concomitant medications and treatment adherence

Across all regions, ICS/LABA users were most commonly prescribed a medium total daily dose of ICS (40.5–54.6%), while high total daily doses of ICS were more commonly prescribed among ICS/LABA maintenance users versus MART users in all regions except China (Supplementary Table 1).

The most commonly co-prescribed asthma medication for all ICS/LABA users in Canada, Europe, and the US was SABA (61.8%, 45.0%, and 67.8%, respectively) (Supplementary Table 1). Co-prescription of SABA was consistently higher among ICS/LABA maintenance versus MART users, except in Japan, where SABA prescription was similar between maintenance and MART users (Fig. 2). For all ICS/LABA users in Japan, leukotriene receptor antagonist (LTRA) prescriptions were most common (32.6%), while in China, long-acting muscarinic antagonists (LAMA) were the most frequently co-prescribed medication (45.6%) (Supplementary Table 1). When stratified by MART and maintenance subpopulations, in Japan, LTRA and LAMA were more commonly co-prescribed among ICS/LABA MART users (40.4% and 10.5%, respectively) than maintenance users (30.8% and 5.7%, respectively), while oral corticosteroids (OCS) were more commonly co-prescribed in maintenance versus MART users (5.2% vs. 3.5%) (Supplementary Table 1). In China, ICS/LABA maintenance users were more likely to receive LAMA (49.2%) or LTRA (18.1%) than MART users (40.4% and 8.5%, respectively) (Supplementary Table 1).

In Canada, Europe, Japan and the US, >90% of all ICS/LABA users were reported as being moderately–completely adherent to their currently prescribed asthma treatment by their treating physician, while in China, most patients were reported as moderately (40.0%) or very adherent (47.2%) (Supplementary Table 1). When stratified by subpopulation, the proportion of patients classed as moderately–completely adherent was broadly similar between ICS/LABA maintenance and MART users in all regions, though there was some variation between subpopulations (Supplementary Table 1).

#### Prescribing patterns and physicians’ reasons for treatment choice

In Canada, Europe, Japan, and the US, treating physicians were most commonly PCPs, followed by pulmonologist/respiratory specialists; allergists made up the smallest proportion of specialists sampled. In China, all physicians were hospital-based specialists (Table 1).

For all ICS/LABA users, the most common reasons physicians gave for their choice of currently prescribed ICS/LABA treatment was to provide sustained 24-hour symptom relief and improve shortness of breath in Canada (57.7%, 58.4%), Europe (60.8%, 57.9%), and Japan (57.9%, 40.3%). In China, improvement in daytime symptomatic relief was prioritized together with improvements in shortness of breath (91.6%, 64.5%), while in the US, sustained 24-hour symptom relief and improvement in daytime symptomatic relief were prioritized (68.2%, 63.3%). When stratified by subpopulation, the key reasons for ICS/LABA MART prescriptions were immediate onset of action in Canada (62.5%) and Europe (60.7%), improvement in daytime symptomatic relief in China (86.5%), improvement in shortness of breath in Japan (54.4%) and sustained 24-hour symptom relief in the US (78.8%). The main reason for ICS/LABA maintenance-only prescriptions was sustained 24-hour symptom relief in Canada (62.2%), Europe (63.3%), Japan (62.1%), and the US (66.1%), whereas improvement in daytime symptomatic relief remained the main reason in China (91.6%).

### Clinical burden

Based on available data, the mean (SD) pre-bronchodilator FEV_1_ % predicted was highest among all ICS/LABA users in Canada (91.2 [109.0]) compared with all other regions. China had the lowest mean (SD) number of exacerbations of any severity (0.2 [0.6]) and exacerbations resulting in an OCS course and/or ER visit and/or hospitalization in the last 12 months (0.1 [0.4]), compared with all other regions (Table 2). The mean (SD) number of exacerbations of any severity in the last 12 months was greater among ICS/LABA MART users versus maintenance users in Canada, Europe, Japan, and the US (Canada: 1.0 [1.2] vs. 0.8 [1.7]; Europe: 0.8 [1.2] vs. 0.7 [1.2]; Japan: 0.7 [1.4] vs. 0.4 [1.2]; US: 1.0 [2.0] vs. 0.8 [1.2]), but remained similar in China (China: 0.2 [0.6] vs. 0.2 [0.6]).

**Fig. 3:**
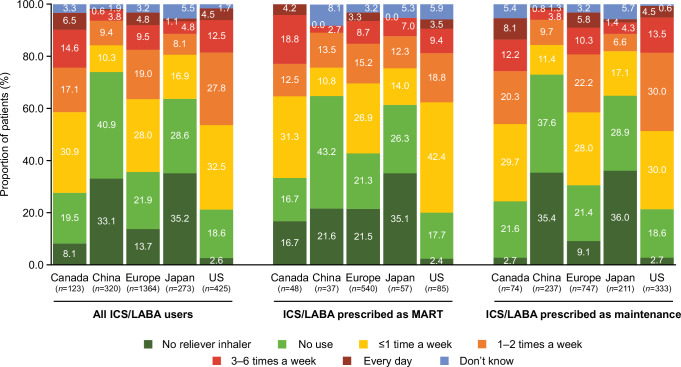
Physician-reported short-acting reliever medication use. ICS inhaled corticosteroid, LABA long-acting β_2_-agonist, MART maintenance and reliever therapy, US United States.

Physician-reported SABA use varied between regions (Fig. 3). The proportion of patients with any SABA use was considerably higher in Canada (69.1%), Europe (61.3%), and the US (77.2%) compared with China (24.1%) and Japan (30.8%); most patients in China and Japan received no SABA or did not use their prescribed SABA. Everyday use was low throughout all regions, ranging from 0.6%–6.5%. When stratified by subpopulation, the proportion of patients with any SABA use was higher among ICS/LABA maintenance versus MART users in Canada (70.3% vs. 66.7%), Europe (66.3% vs. 54.1%) and the US (78.1% vs 74.1%), but was higher among MART versus maintenance users in China (27.0% vs. 25.7%) and Japan (33.3% vs. 29.4%).

### Economic burden

#### Healthcare resource utilization

ICS/LABA users in Japan had the highest mean (SD) number of unscheduled HCP visits (9.6 [4.9]), whereas China (3.1 [2.7]), Europe (2.4 [1.9]), and the US (2.3 [2.1]) had the highest number of scheduled HCP visits compared with other regions (Table 3). A higher mean (SD) number of hospitalizations due to asthma were reported in Europe and the US (each 0.3 [0.7]) compared with Canada (0.1 [0.5]), China (0.1 [0.4]), and Japan (0.1 [0.5]) (Table 3). There were no differences in HCRU between those receiving ICS/LABA as MART or as maintenance in any region (Table 3).

**Table 3:** HCRU in the last 12 months for all ICS/LABA users and MART and maintenance subpopulations.

	All ICS/LABA users					ICS/LABA prescribed as MART					ICS/LABA prescribed as maintenance				
	Canada (*N* = 123)	China (*N* = 321)	Europe (*N* = 1364)	Japan (*N* = 273)	US (*N* = 425)	Canada (*n* = 48)	China (*n* = 37)	Europe (*n* = 540)	Japan (*n* = 57)	US (*n* = 85)	Canada (*n* = 74)	China (*n* = 237)	Europe (*n* = 747)	Japan (*n* = 211)	US (*n* = 333)
Mean (SD) number of HCP visits per patient in last 12 months for any condition	4.6 (4.9)	5.1 (3.4)	4.3 (3.4)	10.4 (5.2)	3.6 (3.0)	4.2 (3.5)	5.2 (4.7)	4.3 (3.8)	11.2 (5.4)	3.4 (2.9)	4.8 (5.7)	5.2 (3.0)	4.2 (3.1)	10.1 (4.9)	3.7 (3.1)
Mean (SD) number of HCP visits per patient in last 12 months for asthma (unscheduled)	*n* = 123 1.6 (1.2)	*n* = 305 1.9 (1.9)	*n* = 1364 0.8 (1.7)	*n* = 273 9.6 (4.9)	*n* = 425 0.4 (0.8)	*n* = 48 1.4 (1.1)	*n* = 31 1.9 (1.3)	n=540 0.9 (2.0)	*n* = 57 9.7 (4.7)	*n* = 85 0.5 (0.9)	*n* = 74 1.7 (1.3)	*n* = 228 2.0 (2.0)	n=747 0.7 (1.3)	*n* = 211 9.5 (4.8)	*n* = 333 0.4 (0.8)
Mean (SD) number of HCP visits per patient in last 12 months for asthma (scheduled)	*n* = 123 0.8 (1.3)	*n* = 319 3.1 (2.7)	*n* = 1364 2.4 (1.9)	*n* = 273 0.6 (1.4)	*n* = 425 2.3 (2.1)	*n* = 48 0.8 (0.8)	*n* = 36 3.2 (3.3)	n=540 2.3 (1.9)	*n* = 57 1.0 (1.8)	*n* = 85 2.1 (1.2)	*n* = 74 0.9 (1.5)	*n* = 228 3.2 (2.7)	*n* = 747 2.5 (1.9)	n=211 0.5 (1.2)	*n* = 333 2.3 (2.3)
Mean (SD) number of hospitalizations per patient in the last 12 months due to asthma, including ER visits and overnight hospitalizations	0.1 (0.5)	0.1 (0.4)	0.3 (0.7)	0.1 (0.5)	0.3 (0.7)	0.1 (0.4)	0.1 (0.5)	0.3 (0.7)	0.1 (0.5)	0.3 (0.7)	0.1 (0.6)	0.1 (0.4)	0.3 (0.7)	0.1 (0.5)	0.3 (0.7)

*ER* emergency room, *HCP* healthcare professional, *HCRU* healthcare resource utilization, *ICS* inhaled corticosteroid, *LABA* long-acting β_2_-agonist, *MART* maintenance and reliever therapy, *SD* standard deviation, *US* United States.

#### Work and activity impairment

**Fig. 4:**
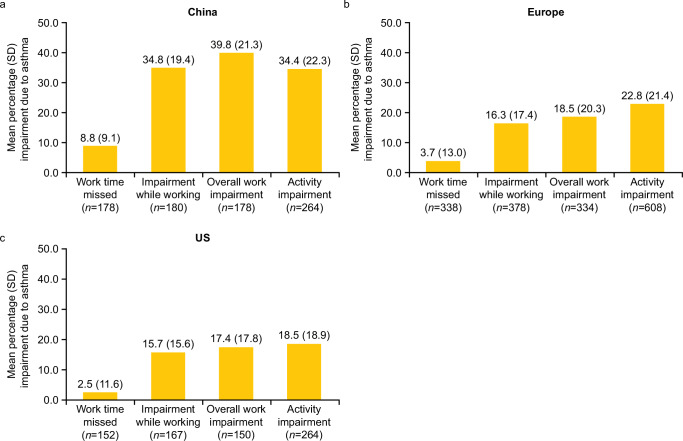
WPAI of all ICS/LABA users. **a** China, **b** Europe, and **c** the US. Data for Canada and Japan were not collected. ICS inhaled corticosteroid, LABA long-acting β_2_-agonist, SD standard deviation, US United States, WPAI Work Productivity and Activity Impairment.

Among all ICS/LABA users with available data, the proportion of patients in full-time employment at baseline ranged from 67.0% to 42.9% across all regions (Table 1). In the China, Europe, and US regions, mean percentage (SD) overall work impairment ranged from 39.8% (21.3) to 17.4% (17.8), with absenteeism ranging from 8.8% (9.1) to 2.5% (11.6) (Fig. 4).

### Humanistic burden

#### Symptom burden

ICS/LABA users in Canada and the US had the highest mean (SD) number of physician-reported symptom-free days in the past 30 days (20.6 [8.9] and 19.7 [8.6], respectively). Similar results were observed for patient-reported symptom-free days in China, Europe, Japan, and the US (Table 4). The most troublesome symptoms among all ICS/LABA users varied across regions, and included shortness of breath when exposed to a trigger (Canada [20.3%]), productive cough (China [28.0%] and Japan [18.0%]), and shortness of breath during exertion (Europe [33.4%] and the US [35.5%]) (Table 4).

**Table 4:** Symptom burden for all ICS/LABA users.

	All ICS/LABA users				
	Canada (N=123)	China (N=321)	Europe (N=1,364)	Japan (N=273)	US (N=425)
**Physician-reported number of symptom-free days in the past 30 days**	*n* = 97	*n* = 320	*n* = 1020	*n* = 183	*n* = 317
Mean (SD)	20.6 (8.9)	10.1 (9.3)	17.9 (9.2)	18.8 (9.5)	19.7 (8.6)
**Patient-reported number of symptom-free days in the past 30 days**		*n* = 321	*n* = 569	*n* = 94	*n* = 201
Mean (SD)	N/A	10.0 (9.0)	18.3 (10.0)	19.1 (10.5)	19.6 (9.5)
**Physician-reported most troublesome symptoms experienced in the last 12 months, %**	*n* = 123	*n* = 296	*n* = 990	*n* = 273	*n* = 310
Dry cough	5.7	18.9	15.6	5.1	9.4
Productive cough	11.4	28.0	4.3	18.0	5.5
Regular clearing of throat	5.7	2.4	2.2	13.2	2.6
Shortness of breath during exertion	4.1	15.9	33.4	5.9	35.5
Shortness of breath when exposed to trigger	20.3	14.5	15.9	11.7	11.6
Tight feeling in the chest	0.8	4.7	4.2	2.2	6.1
Wheezing	16.3	7.1	9.7	1.5	16.8
None of the above	31.7	2.7	1.2	40.3	2.6

Data for variables indicated as N/A were not collected.

*ICS* inhaled corticosteroid, *LABA* long-acting β_2_-agonist, *N/A* not available, *SD* standard deviation, *US* United States.

#### Health-related quality of life

**Fig. 5:**
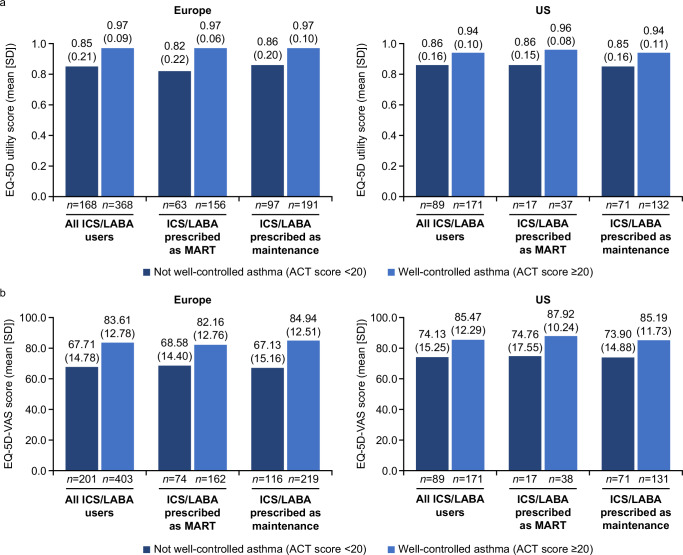
EQ-5D utility and EQ-5D-VAS scores stratified by asthma control status and treatment group. **a** EQ-5D utility and **b** EQ-5D-VAS scores. Data for Canada, China, and Japan were not collected. A score of 1.0 for EQ-5D utility and of 100.0 for EQ-5D-VAS denote the best health state. ACT Asthma Control Test, EQ-5D EuroQol 5 dimensions, ICS inhaled corticosteroid, LABA long-acting β_2_-agonist, MART maintenance and reliever therapy, SD standard deviation, US United States, VAS visual analog scale.

Among patients with available data in Europe and the US, approximately 1 in 3 patients in all subpopulations reported having not well-controlled asthma (Europe: 31.5–34.6%; US: 30.9–35.1%) (Supplementary Fig. 2A). The impact of asthma on sleep was also similar across all subpopulations, with mean JSEQ scores ranging from 3.0–3.4 in Europe and 2.9–3.6 in the US (Supplementary Fig. 2B). When stratified by asthma control status, mean EQ-5D utility scores and EQ-5D-VAS scores in Europe and the US were numerically lower across all treatment subpopulations for patients with not well-controlled asthma versus patients with well-controlled asthma (EQ-5D utility: Europe, 0.82–0.86 vs. 0.97; US, 0.85–0.86 vs. 0.94–0.96; EQ-5D-VAS: Europe, 67.13–68.58 vs. 82.16–84.94; US, 73.90–74.76 vs. 85.19–87.92) (Fig. 5).

## Discussion

This study showed that patients receiving ICS/LABA as maintenance or MART share similar demographic and clinical characteristics in Canada, China, Europe, Japan, and the US. Despite most patients being classed as moderately–completely adherent to their ICS/LABA regimen by their physician, the results demonstrate that patients in all 5 regions still experience clinical, economic, and humanistic impairment as a result of their asthma. For example, most patients were at GINA treatment Step 4, up to 40% had experienced an exacerbation in the year prior to the survey date, and patients from all regions had both unscheduled and scheduled HCP visits due to their asthma. In China, Europe, and the US, ICS/LABA users reported a mean percentage of work impairment due to asthma of 15.7–39.8% across most WPAI domains. In addition, ICS/LABA users in Canada, Europe, Japan, and the US experienced symptoms on approximately 31–40% of days in the past 30 days, while approximately one-third of ICS/LABA users in Europe and the US had not well-controlled asthma (defined by an ACT score <20), with similar rates between MART and maintenance users in both regions. The WPAI, asthma control, and symptom burden data reported here broadly correspond to a 2018 US study by Davis et al. ^10^, and are consistent with previous studies showing that poor asthma control is associated with symptomatic, economic, and humanistic burden^10,17–19^.

Despite the recommendation of ICS/LABA as MART for those at GINA treatment Step 3 and above^1^, this study showed that ICS/LABA MART usage across the regions remains low (13.4–39.6%) compared with ICS/LABA as maintenance (54.8–78.4%). This is consistent with a 2021 study by Chapman et al., where MART prescription was found to be between 7.8% and 14.9% among patients from Australia, Canada, China, and the Philippines, with maintenance ICS or ICS/LABA with SABA the most widely used regimens^20^. This could suggest that there is an unmet need for increased awareness of MART by prescribers. In addition, although there was variation across regions in physician reason for prescribing ICS/LABA as either MART or maintenance, the focus overall was in reduction of symptom burden, rather than a reduction in risk of exacerbations. This could further highlight the need for increased education around the possible benefits of ICS/LABA as MART, however, further research is needed to investigate the reasoning behind physicians’ decisions.

Poor asthma control is also linked to an increased use of reliever therapy^1^. The GINA 2024 report identifies the use of as-needed SABA more than twice a week as an indicator for inadequately controlled asthma^1^. In this study, 33.3–44.7% of ICS/LABA users in Canada, Europe, and the US used SABA at least once a week, and 1 in 3 ICS/LABA users in Europe and the US had not well-controlled asthma. In line with our results, a previous study of 744 patients attending pulmonary and allergy clinics in the US showed that 45.7% (low ICS dose) to 59.7% (high ICS dose) of ICS/LABA users reported that their asthma was not well-controlled^18^. These patients had worse HRQoL, greater exacerbation burden and HCRU, and greater use of systemic corticosteroids than patients with well-controlled asthma^18^. Moreover, a 2022 study by Vähätalo et al., found that high SABA use was associated with higher-dose ICS prescriptions^21^. Here, we observed that 26.6–33.9% of patients were prescribed a high total daily dose of ICS. In the context of the findings by Vähätalo et al.^21^, this suggests that ICS/LABA prescriptions with high-dose ICS may be associated with the high levels of SABA use reported here, although further research is needed to explore this link. Collectively, these results highlight the need for additional treatments to control asthma symptoms and reduce the use of short-acting reliever medication. For patients with uncontrolled moderate-to-severe asthma, GINA recommends add-on LAMA as either multiple- or single-inhaler ICS/LAMA/LABA triple therapy^1^, which has been shown to improve lung function, reduce exacerbations, and improve asthma control in patients with inadequately controlled or uncontrolled asthma despite ICS/LABA therapy^22–24^.

This study also showed that 5–25% of patients across all five regions reported taking SABA medication 3 or more times a week, suggesting that there is a subset of patients receiving ICS/LABA who may be overusing their reliever medication. It should be noted that the proportion of patients using SABA 3 or more times a week in this study is lower than that reported in a recent real-world SABINA study reporting SABA overuse in Canada (49–61%)^5^. However, SABA overuse in the SABINA study was analyzed using administrative claims data, which can confirm prescription dispensation but cannot confirm actual SABA use^5^. In comparison, data for the frequency of SABA use presented here were reported by the treating physician, which may provide a more accurate real-world representation of patients’ actual SABA use. Furthermore, we found that levels of SABA co-prescribing were higher among ICS/LABA maintenance users versus MART users in Canada, China, Europe, and the US. This is unsurprising given that SABA is recommended as-needed alongside ICS/LABA maintenance prescriptions^1^. However, we also observed that SABA co-prescribing remained relatively high among ICS/LABA MART users in Canada, Europe, Japan, and the US, and that mean number of exacerbations were also higher among ICS/LABA MART users in these regions than among ICS/LABA maintenance users. This is notable considering that ICS/LABA in this population should be used as reliever medication^1^, and given that SABA usage is associated with reduced asthma control and increased exacerbation risk^25–27^. Together, these findings suggest potentially inappropriate ICS/LABA prescribing as MART and/or a continued and potentially detrimental over-reliance on additional short-acting reliever medication on top of ICS/LABA MART.

This study has a number of strengths. The data reported here provide valuable insight into the current understanding of asthma management in Canada, China, Europe, Japan, and the US, using an established methodology that reflects the real-world care of patients with asthma. The inclusion of a patient self-completion questionnaire allowed a holistic perspective of asthma burden and was not wholly reliant on, nor fully matched for responses from the physician perspective. In addition, the DSP survey methodology identified MART prescribing by prompting physicians to respond via a tick box on how ICS/LABA had been prescribed. As such, the DSP survey collected complete ICS/LABA prescribing data rather than limited or missing prescribing instructions captured as part of electronic medical records in secondary data sources.

There are also several limitations that should be considered when interpreting these results. No formal statistical analyses were performed in this study, therefore, all comparisons between regions and subpopulations were descriptive. The survey reflected the study populations over different time periods in each region, which may impact comparisons across different markets, for example, guideline recommendations in the different regions may have been updated in this time, and as such, data were unable to be combined to provide a global view of ICS/LABA usage. Furthermore, the availability and use of PRO instruments differed between countries; WPAI data were not collected for Canada and Japan, while ACT and JSEQ data were not collected for Canada, China, and Japan. As such, PRO comparisons across all regions were sometimes limited. The number of patients prescribed MART was smaller than those prescribed maintenance-only in all regions, but as these two subpopulations were compared descriptively, no causal relationships between treatment and outcomes can be determined. In addition, patients participating in the Asthma DSP may not have reflected the general asthma population, and the DSP was not based on a true random sample of physicians or patients. Participation was voluntary and influenced by pragmatic geographical considerations such as a concentration of recruitment in urban areas. Moreover, no formal procedures were in place for verifying patient selection, with identification of the target patient group based on the judgement of the treating physician, although the requirement for physicians to provide data for a consecutive patient sample aimed to reduce selection bias. A further limitation was the challenge of evaluating whether ICS/LABA was being used as prescribed, despite physician confirmation of intended use of the prescribed therapy. Similarly, there are challenges associated with assessing patient adherence via survey to physicians, as this is a subjective measure that may be open to bias in not being directly posed to patients. Lastly, recall bias is a common limitation of surveys and, therefore, may have affected the questionnaire responses from patients and physicians. However, as the data for these analyses were captured during the patient’s appointment, this is expected to have reduced the likelihood of recall bias.

In conclusion, patients with asthma continued to experience clinical and symptomatic burden despite receiving ICS/LABA as daily MART or maintenance-only therapy, which led to work and activity impairment and impacted patients’ HRQoL. These findings add valuable insights and evidence to the current understanding of asthma management within a multinational context and highlight the continued burden of disease among ICS/LABA users, irrespective of treatment modality.

## Supplementary information

Supplementary Materials

Supplementary Figure 1

Supplementary Figure 2

## Data Availability

The Adelphi Real World Asthma DSP was used under license from Adelphi Real World and is not publicly available. Requests for anonymized participant data can be made via a request to the corresponding author (S.Z.) and study documents for the parent study can be requested for further research from https://www.gsk-studyregister.com/en/.

## Abbreviations

ACT: Asthma Control Test; DSP, Disease Specific Program
FeNO: fractional exhaled nitric oxide; EQ-5D, EuroQol 5 dimensions
FEV_1_: forced expiratory volume in 1 second
GERD: gastroesophageal reflux disease; GINA, Global Initiative for Asthma
HCP: healthcare professional
HCRU: healthcare resource utilization
HRQoL: health-related quality of life
ICS: inhaled corticosteroids
JSEQ: Jenkins Sleep Evaluation Questionnaire
LABA: long-acting β_2_-agonist
LAMA: long-acting muscarinic antagonist
LTRA: leukotriene receptor antagonist
MART: maintenance and reliever therapy
OCS: oral corticosteroids; PCP, primary care physician
PDE-4: phosphodiesterase-4
PRO: patient-reported outcome
SABA: short-acting β_2_-agonist
SAMA: short-acting muscarinic antagonist
WPAI: Work Productivity and Activity Impairment.
