# Supplementary material for: Burden of illness among patients with asthma prescribed inhaled corticosteroids/long-acting β2-agonists
Source: NPJ Prim Care Respir Med. 2025 Feb 26;35:10. doi: 10.1038/s41533-024-00402-w (PMC11865622; doi:10.1038/s41533-024-00402-w)
Supplement: Supplementary file 1 — Supplementary Materials [file 41533_2024_402_MOESM1_ESM.pdf]

Burden of illness among patients with asthma prescribed inhaled corticosteroids/long-acting β_2-agonists

Supplementary Material

Supplementary Figure 1. Identification of MART prescribing

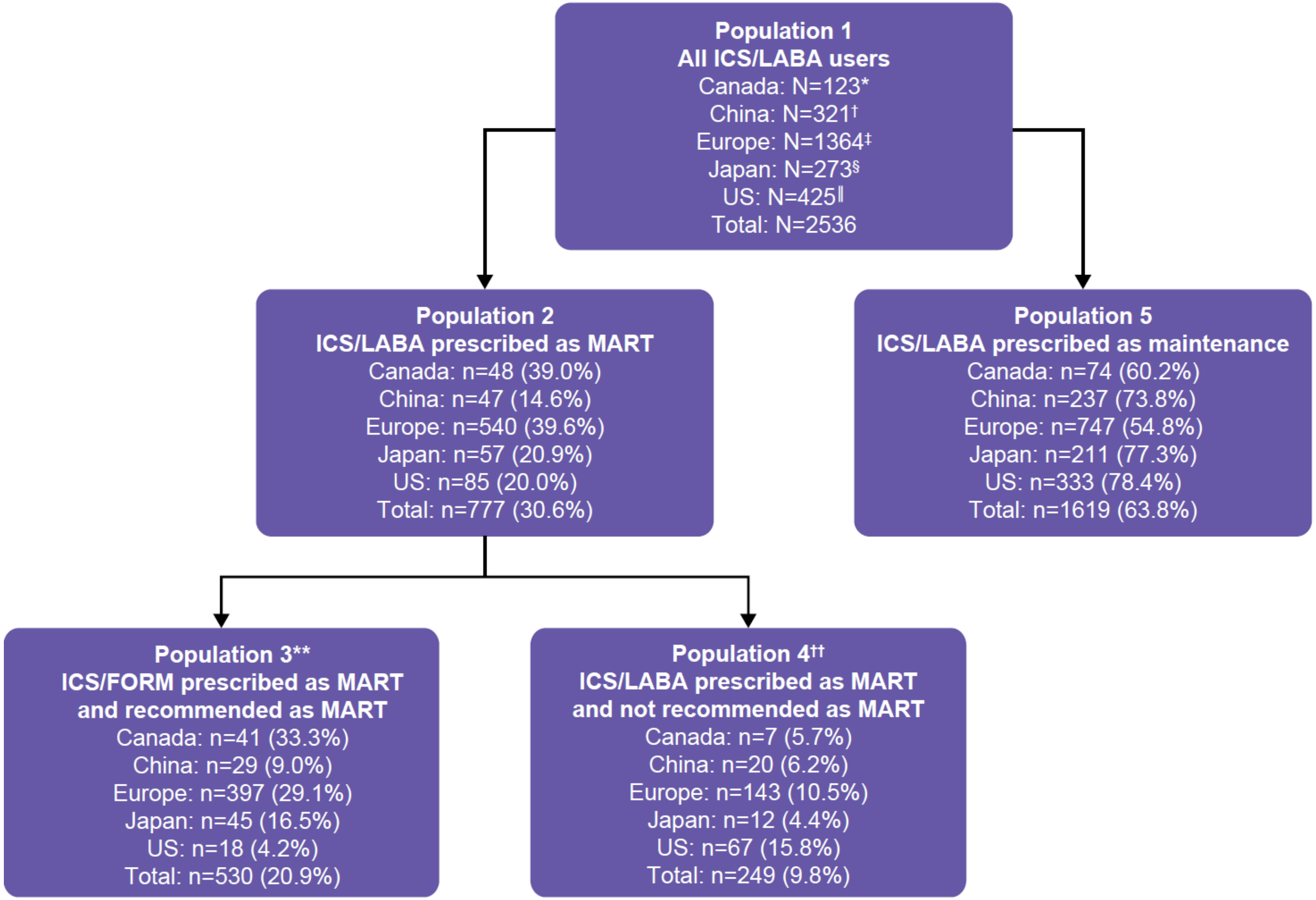

Percentage values are given as the percentage of Population 1 for each country or total category.

*1 patient was missing as they were currently receiving ICS/LABA as reliever only therapy; [†]47 patients were missing as they were currently receiving ICS/LABA as reliever only therapy; [‡]77 patients were missing as they were currently receiving ICS/LABA as reliever only therapy; [§]5 patients were missing as they were currently receiving ICS/LABA as reliever only therapy; [‖]7 patients were missing as they were currently receiving ICS/LABA as reliever only therapy; **Includes beclomethasone/formoterol and budesonide/formoterol; [††]All ICS/LABA excluding beclomethasone/formoterol and budesonide/formoterol.

FORM, formoterol; ICS, inhaled corticosteroid; LABA, long-acting β_2-agonist; MART, maintenance and reliever therapy; US, United States.

*Supplementary Figure 2. Asthma control status (a) and JSEQ scores (b)**

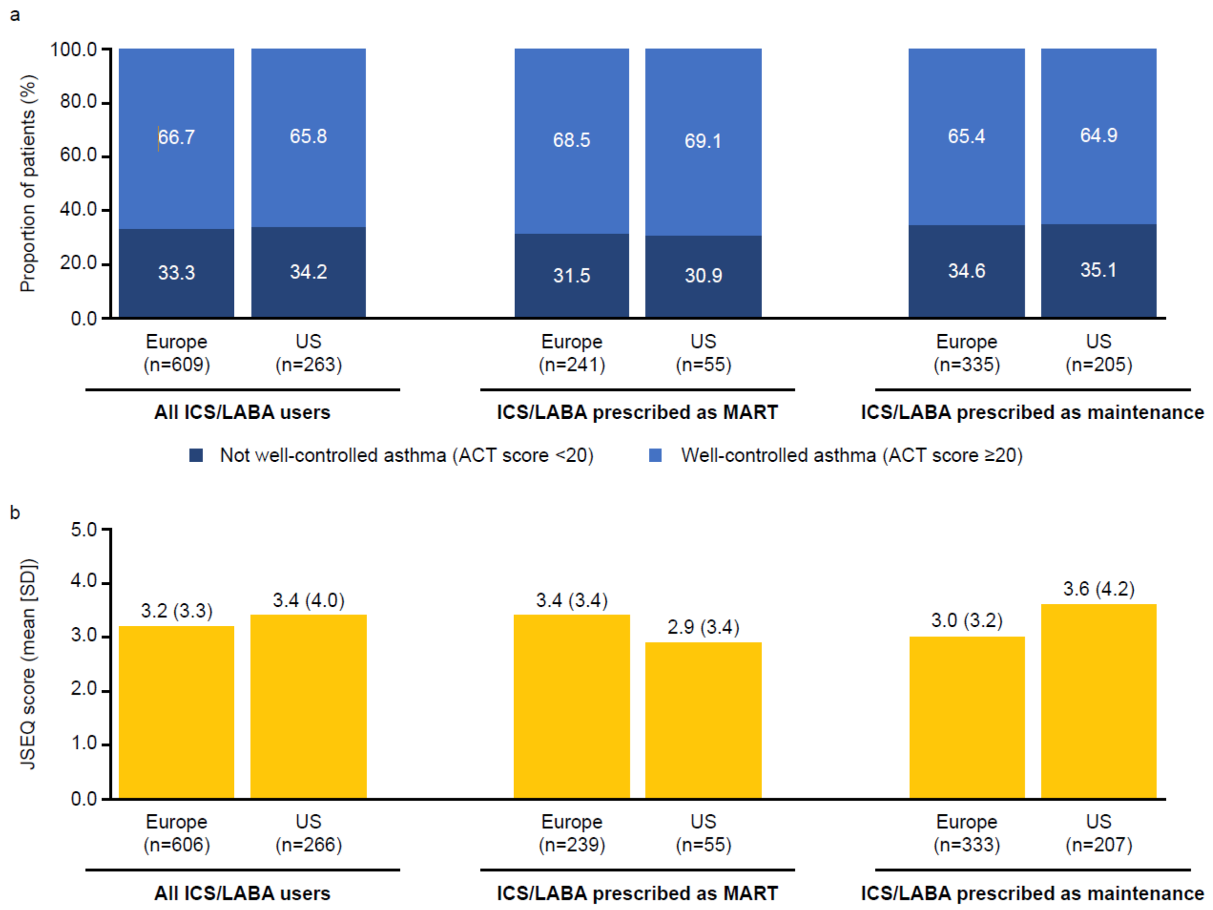

*Data for Canada, China, and Japan were not collected.

ACT, Asthma Control Test; ICS, inhaled corticosteroid; JSEQ, Jenkins Sleep Evaluation Questionnaire; LABA, long-acting β_2-agonist; MART, maintenance and reliever therapy; SD, standard deviation; US, United States.

Supplementary Table 1. Currently prescribed asthma treatments and physician-reported adherence in all ICS/LABA users, and MART and maintenance subpopulations

	All ICS/LABA users					ICS/LABA prescribed as MART					ICS/LABA prescribed as maintenance				
	Canada (N=123)	China (N=321)	Europe (N=1,364)	Japan (N=273)	US (N=425)	Canada (n=48)	China (n=37)	Europe (n=540)	Japan (n=57)	US (n=85)	Canada (n=74)	China (n=237)	Europe (n=747)	Japan (n=211)	US (n=333)
**Current ICS total daily dose, %[1]**	*n=113*	*n=181*	*n= 1,325*	*n=263*	*n=401*	*n=47*	*N=18*	*n=524*	*n=55*	*n=82*	*n=65*	*N=150*	*n=728*	*n=204*	*n=312*
Low	21.2	40.9	34.5	25.5	20.2	34.0	50.0	43.5	32.7	22.0	10.8	38.0	28.3	23.0	18.9
Medium	54.0	50.8	40.5	43.4	54.6	53.2	33.3	36.1	45.5	58.5	55.4	54.7	43.5	43.1	54.5
High	24.8	8.3	25.1	31.2	25.2	12.8	16.7	20.4	21.8	19.5	33.9	7.3	28.2	33.8	26.6
**Currently prescribed asthma treatment, %**															
ICS	0.8	1.7	2.3	0.7	1.4	0.0	6.4	3.0	0.0	0.0	1.4	1.2	1.5	1.0	1.8
OCS	2.4	0.3	3.0	5.1	2.4	4.2	0.0	4.1	3.5	1.2	1.4	0.4	2.1	5.2	2.7
LABA	0.8	0.0	0.6	0.7	0.0	2.1	0.0	1.1	0.0	0.0	0.0	0.0	0.0	1.0	0.0
LAMA	11.4	45.6	7.5	7.0	3.5	16.7	40.4	8.0	10.5	4.7	6.7	49.2	7.1	5.7	3.3
LABA/LAMA	0.0	0.0	0.1	0.0	0.2	0.0	0.0	0.0	0.0	1.2	0.0	0.0	0.1	0.0	0.0
PDE-4 inhibitors	0.0	0.0	0.1	0.0	0.2	0.0	0.0	0.0	0.0	0.0	0.0	0.0	0.1	0.0	0.3
Biologics	4.1	0.0	2.9	1.5	4.7	4.2	0.0	2.6	0.0	5.9	4.1	0.0	3.2	1.9	4.5
SABA	61.8	23.4	45.0	8.8	67.8	33.3	6.4	24.4	8.8	52.9	79.7	25.6	61.3	8.5	71.8
SAMA	0.8	2.6	1.2	0.0	1.9	0.0	2.1	0.7	0.0	4.7	1.4	3.2	1.3	0.0	0.9

SABA/SAMA	0.0	2.3	0.5	1.1	1.2	0.0	4.3	0.0	1.8	1.2	0.0	1.6	0.9	1.0	1.2
ICS/LABA/LAMA	0.0	0.0	0.3	0.0	0.5	0.0	0.0	0.0	0.0	1.2	0.0	0.0	0.1	0.0	0.0
LTRA	14.6	17.4	13.1	32.6	24.9	20.8	8.5	14.6	40.4	29.4	10.8	18.1	12.2	30.8	24.3
**Physician-reported adherence, %[2]**	*n=123*	*n=320*	*n= 1,364*	*n=273*	*n=425*	*n=48*	*n=37*	*n=540*	*n=57*	*n=85*	*n=74*	*n=236*	*n=747*	*n=211*	*n=333*
Not at all	1.6	0.0	0.4	0.7	1.2	2.1	0.0	0.4	0.0	0.0	1.4	0.0	0.4	1.0	1.2
Slightly	3.3	6.6	3.0	4.4	4.0	6.3	13.5	3.9	1.8	2.4	1.4	5.9	2.7	5.2	4.2
Moderately	23.5	40.0	21.8	26.7	18.4	16.7	37.8	25.2	29.8	18.8	28.4	37.3	18.7	24.6	18.6
Very	46.3	47.2	40.6	45.4	35.3	56.3	37.8	40.4	35.1	40.0	39.2	50.9	40.6	48.8	33.9
Completely	25.2	6.3	34.2	22.7	41.2	18.8	10.8	30.2	33.3	38.8	29.7	5.9	37.6	20.4	42.0

[1]Ranges for ICS total daily doses were derived from GINA 2018 recommendations[30]; [2]physician-reported patient adherence was measured by a single choice question in the patient record form: "How adherent is this patient with their treatment regimen in terms of the number of times they take their asthma treatment as prescribed in the last 12 months?" Responses were scored using a 5-point scale ranging from 'not at all' adherent to 'completely' adherent.

GINA, Global Initiative for Asthma; ICS, inhaled corticosteroid; LABA, long-acting β_2-agonist; LAMA, long-acting muscarinic antagonist; LTRA, leukotriene receptor antagonist; MART, maintenance and reliever therapy; OCS, oral corticosteroid; PDE-4, phosphodiesterase-4; SABA, short-acting β_2-agonist; SAMA, short-acting muscarinic antagonist; US, United States.
